# Understanding Galectin-3’s Role in Diastolic Dysfunction: A Contemporary Perspective

**DOI:** 10.3390/life14070906

**Published:** 2024-07-20

**Authors:** Wen-Rui Hao, Chun-Han Cheng, Ju-Chi Liu, Huan-Yuan Chen, Jin-Jer Chen, Tzu-Hurng Cheng

**Affiliations:** 1 Division of Cardiology, Department of Internal Medicine, Shuang Ho Hospital, Ministry of Health and Welfare, Taipei Medical University, New Taipei City 23561, Taiwan; b8501043@tmu.edu.tw (W.-R.H.); liumdcv@tmu.edu.tw (J.-C.L.); 2 Division of Cardiology, Department of Internal Medicine, School of Medicine, College of Medicine, Taipei Medical University, Taipei City 11002, Taiwan; 3 Department of Medical Education, Linkou Chang Gung Memorial Hospital, Taoyuan City 33305, Taiwan; leocheng1991@gmail.com; 4 Institute of Biomedical Sciences, Academia Sinica, Taipei 11529, Taiwan; hchen9@ibms.sinica.edu.tw (H.-Y.C.); jc8510@yahoo.com (J.-J.C.); 5 Division of Cardiology, Department of Internal Medicine and Graduate Institute of Clinical Medical Science, China Medical University, Taichung City 404333, Taiwan; 6 Department of Biochemistry, School of Medicine, College of Medicine, China Medical University, Taichung City 404333, Taiwan

**Keywords:** diastolic dysfunction, galectin-3, fibrosis, biomarker, heart failure, cardiac remodeling

## Abstract

Diastolic dysfunction, a prevalent condition characterized by impaired relaxation and filling of the left ventricle, significantly contributes to heart failure with preserved ejection fraction (HFpEF). Galectin-3, a β-galactoside-binding lectin, has garnered attention as a potential biomarker and mediator of fibrosis and inflammation in cardiovascular diseases. This comprehensive review investigates the impact of galectin-3 on diastolic dysfunction. We explore its molecular mechanisms, including its involvement in cellular signaling pathways and interaction with components of the extracellular matrix. Evidence from both animal models and clinical studies elucidates galectin-3’s role in cardiac remodeling, inflammation, and fibrosis, shedding light on the underlying pathophysiology of diastolic dysfunction. Additionally, we examine the diagnostic and therapeutic implications of galectin-3 in diastolic dysfunction, emphasizing its potential as both a biomarker and a therapeutic target. This review underscores the significance of comprehending galectin-3’s role in diastolic dysfunction and its promise in enhancing diagnosis and treatment approaches for HFpEF patients.

## 1. Introduction

### 1.1. Overview of Diastolic Dysfunction

Diastolic dysfunction, a hallmark of heart failure with preserved ejection fraction (HFpEF), manifests as impaired left ventricular relaxation and filling. Myofilament dysfunction, impacting cardiomyocyte contractility and myocardial stiffness, is pivotal in the pathogenesis of diastolic heart failure pathogenesis [1]. Early pericyte loss initiates microvascular dysfunction, driving the progression of diastolic dysfunction [2]. Additionally, cardiomyocytes contribute via altered calcium handling and sarcomeric protein isoform shifts [3]. Coronary microvascular dysfunction exacerbates diastolic dysfunction, leading to left ventricular remodeling [4]. These complexities underscore diastolic dysfunction’s multifactorial nature, involving cellular and microvascular components. Understanding these mechanisms is crucial for developing targeted HFpEF therapies.

### 1.2. Introduction to Galectin-3

Galectin-3, a member of the β-galactoside-binding lectin family, plays a pivotal role in cardiovascular pathology. Its relevance in HFpEF as a potential biomarker for diagnosing and prognosticating cardiac dysfunction is well established [5]. Moreover, galectin-3 influences fibrotic, inflammatory, and remodeling pathways across various cardiac conditions, extending beyond HFpEF [6]. Investigations into galectin-3’s mechanisms offer novel therapeutic avenues, including inhibitors, for cardiovascular diseases [7]. Clinical assessments affirm its diagnostic utility and prognostic significance in chronic heart failure [8]. Understanding galectin-3’s multifaceted involvement in cardiovascular diseases is crucial for unlocking its therapeutic and diagnostic potential in managing conditions like diastolic dysfunction.

### 1.3. Rationale for Exploring Galectin-3 in Diastolic Dysfunction

The investigation of galectin-3 in diastolic dysfunction stems from its intricate involvement in cardiovascular pathology and its potential prognostic value. Galectin-3 levels are significantly associated with adverse outcomes across various cardiovascular disorders, notably HFpEF [9]. Emerging evidence links galectin-3 with systemic proinflammatory-profibrotic responses in conditions such as aortic stenosis and diabetes, suggesting its role in myocardial remodeling and prognosis [10]. For instance, Kocyigit et al. highlighted the role of galectin-3 as a marker of thrombogenicity in atrial fibrillation, emphasizing its potential implications in cardiovascular pathophysiology [11]. Furthermore, Yalcin et al. demonstrated a significant association between serum galectin-3 levels and atrial electrical and structural remodeling, providing deeper insights into its involvement in cardiac remodeling processes [12]. Additionally, galectin-3 serves as an early indicator of diastolic dysfunction in pediatric hemodialysis patients, highlighting its potential in early detection [13]. Given the limited treatment options for diastolic dysfunction and galectin-3’s importance in cardiovascular diseases, investigating its role offers promise for enhancing risk assessment and guiding therapeutic strategies.

## 2. Molecular Mechanisms of Galectin-3

### 2.1. Structure and Function of Galectin-3

Understanding the structural and functional intricacies of galectin-3 is paramount for deciphering its myriad roles in physiological and pathological processes, particularly in diastolic dysfunction. Galectin-3 comprises a carbohydrate recognition domain linked to a collagen-like N-terminal domain, facilitating interactions with β-galactosides on glycoproteins and glycolipids, thereby modulating diverse cellular processes [14]. Functionally, galectin-3 participates in clathrin-independent endocytosis by associating with dynein, a microtubule motor protein, thereby facilitating intracellular trafficking crucial for cellular homeostasis [15]. Moreover, galectin-3’s involvement in fibrosis and inflammation underscores its significance in cardiovascular conditions such as cirrhotic cardiomyopathy, contributing to disease pathogenesis and progression [16]. Notably, targeting galectin-3 emerges as a potential therapeutic strategy, with neutralizing antibodies showing promise in mitigating fibrotic processes, as evidenced in systemic sclerosis treatment [17].

### 2.2. Cellular Signaling Pathways

**Figure 1:**
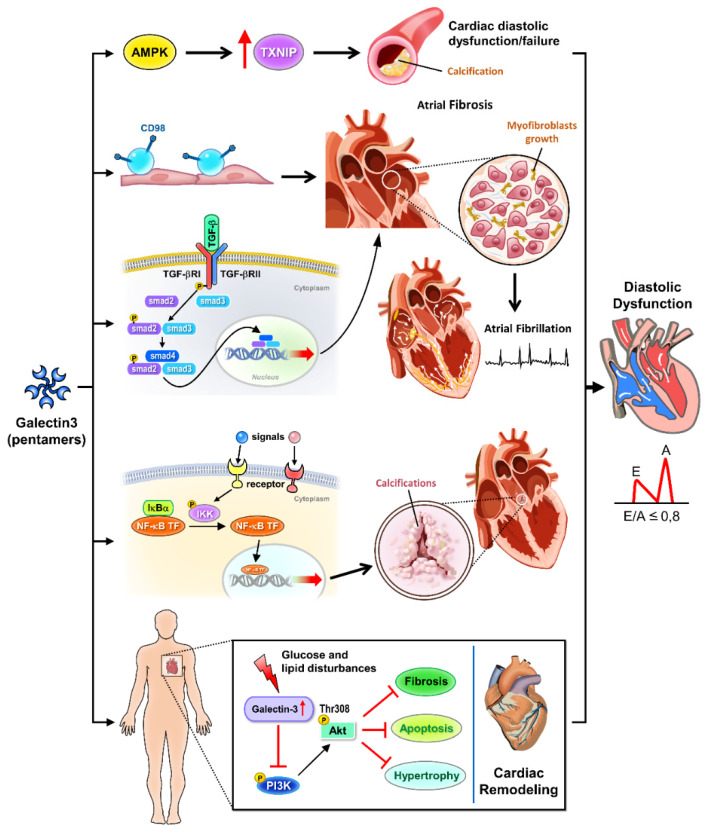
Cellular signaling pathways of galectin-3 in cardiac diastolic pathophysiology.

Galectin-3 intricately modulates cellular signaling pathways implicated in cardiovascular pathophysiology, with ramifications extending to diastolic dysfunction (Figure 1). In vascular calcification, galectin-3 promotes calcification of vascular smooth muscle cells through the AMP-activated protein kinase/thioredoxin-interacting protein pathway, exacerbating arterial stiffness and atherosclerosis progression, crucial features linked to diastolic dysfunction [18]. Furthermore, galectin-3 enhances atrial fibrosis via CD98 signaling, facilitating atrial fibrillation development, a prevalent arrhythmia in diastolic dysfunction patients [19]. Additionally, galectin-3 mediates cardiac remodeling by interfering with glucose and lipid metabolism pathways, hindering Akt activation, and worsening cardiac dysfunction, which may further impair diastolic function [20]. Galectin-3’s involvement in promoting calcification of human aortic valve interstitial cells through the NF-kappa B (nuclear factor kappa-light-chain enhancer of activated B cells) signaling pathway and inducing atrial fibrosis via the TGF beta1 (transforming growth factor beta1)/Smad (suppressor of mother against decapentaplegic) pathway further highlights its role in valvular pathologies and atrial remodeling, impacting diastolic filling [21,22].

Galectin-3 is a key regulator in various signaling pathways that contribute to cardiac diastolic dysfunction. Activation of AMPK and inhibition of TXNIP mitigate calcification processes and glucose and lipid disturbances. Galectin-3 modulates CD98 and TGF-β signaling, promoting atrial fibrosis and myofibroblast growth, which are critical factors in the development of atrial fibrillation. The NF-κB pathway is involved in valve calcification and further contributes to cardiac remodeling. Additionally, galectin-3 influences Akt and PI3K signaling pathways, exacerbating cardiac remodeling and overall diastolic dysfunction. AMPK: AMP-activated protein kinase, involved in metabolic regulation and the inhibition of cardiac calcification. TXNIP: thioredoxin-interacting protein, associated with glucose and lipid disturbances. Calcification: pathological calcification processes in the heart. CD98: cell surface protein influencing TGF-β signaling and fibrosis. TGF-β: transforming growth factor-beta, promoting atrial fibrosis and myofibroblast growth. Atrial fibrosis: fibrotic changes in the atria contributing to atrial fibrillation. Myofibroblast growth: expansion of myofibroblasts, cells involved in fibrosis. Atrial fibrillation: a common cardiac arrhythmia linked to fibrosis. NF-κB: nuclear factor kappa-light-chain enhancer of activated B cells, implicated in inflammation and valve calcification. Valve calcification: calcification affecting heart valves, contributing to cardiac dysfunction. Glucose and lipid disturbance: metabolic disruptions influenced by galectin-3 signaling. Akt: protein kinase B, involved in cell survival and growth signaling pathways. PI3K: phosphoinositide 3-kinase, involved in cell growth and survival signaling. Cardiac remodeling: structural changes in the heart associated with diastolic dysfunction.

### 2.3. Interaction with Extracellular Matrix Components

**Figure 2:**
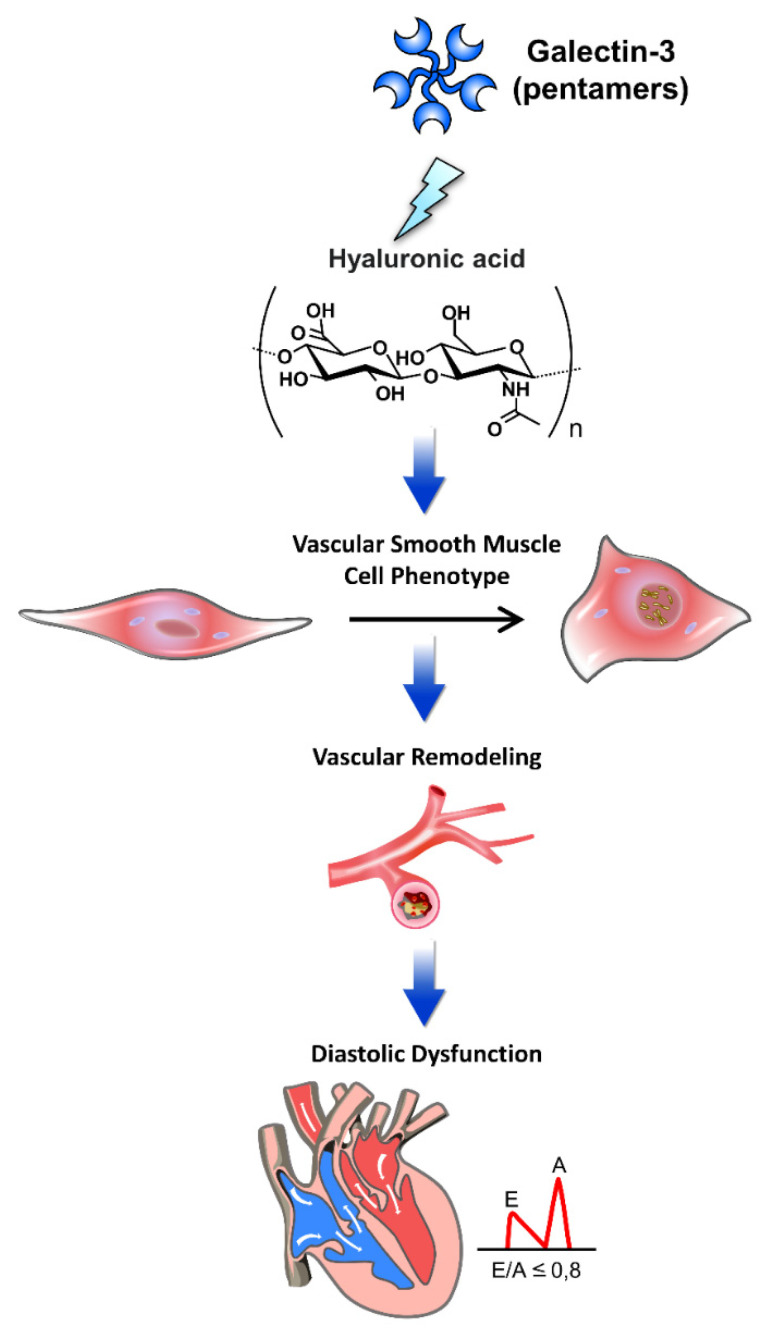
The pathophysiology of galectin-3 interacted with extracellular matrix components in cardiac diastolic dysfunction.

Galectin-3 dynamically interacts with extracellular matrix (ECM) components, exerting profound effects on cardiovascular pathophysiology, including diastolic dysfunction (Figure 2). The study by Nuzzi et al. (2023) highlights galectin-3’s potential role in left atrial (LA) reverse remodeling, suggesting significant prognostic implications [23]. Elevated galectin-3 levels, linked to fibrosis and inflammation, may influence LA structural changes. The study indicates that effective LA reverse remodeling, associated with lower galectin-3 levels, could improve clinical outcomes in patients with dilated cardiomyopathy, underscoring the importance of galectin-3 as a biomarker for therapeutic strategies and prognosis in heart failure management. In dilated cardiomyopathy, circulating galectin-3 levels correlate with ECM fibrosis, suggesting its involvement in cardiac remodeling [24]. Furthermore, galectin-3 reflects echocardiographic quantification of right ventricular failure, indicating its potential as a biomarker for assessing cardiac dysfunction related to ECM alterations [25]. Galectin-3’s interaction with hyaluronan influences the vascular smooth muscle cell phenotype in atherosclerosis and contributes to cardiac macrophage responses following ischemia-reperfusion injury, impacting vascular remodeling and cardiac repair processes relevant to diastolic dysfunction [26,27]. Additionally, diabetes-associated alterations in serum biomarkers, including galectin-3, reflect ECM remodeling and contribute to diastolic dysfunction progression, highlighting the intricate interplay between galectin-3 and ECM dynamics in diabetic cardiomyopathy [28]. Understanding these interactions provides insights into the pathogenesis of diastolic dysfunction and potential therapeutic targets for improving cardiovascular outcomes.

Galectin-3 interacts with various extracellular matrix (ECM) components, playing a pivotal role in the pathophysiology of cardiac diastolic dysfunction. This figure illustrates the mechanisms by which galectin-3 influences cardiac structure and function through its interactions with ECM components. Through these interactions, galectin-3 contributes to the progression of diastolic dysfunction by promoting ECM deposition, vascular remodeling, and alterations in the vascular smooth muscle cell phenotype, leading to increased myocardial stiffness and impaired cardiac function. Hyaluronic acid: an ECM glycosaminoglycan that interacts with galectin-3, contributing to tissue stiffness and fibrosis. Vascular smooth muscle cell phenotype: galectin-3 affects the phenotype of vascular smooth muscle cells, promoting a pro-fibrotic and pro-inflammatory state. Vascular remodeling: structural changes in blood vessels induced by galectin-3, leading to increased stiffness and altered function. Diastolic dysfunction: impaired relaxation and filling of the heart during diastole, exacerbated by galectin-3-mediated ECM alterations and vascular remodeling.

## 3. Role of Galectin-3 in Diastolic Dysfunction

### 3.1. Experimental Evidence from Animal Models

Experimental inquiries into the role of galectin-3 in diastolic dysfunction using animal models have yielded crucial insights. Several key studies provide detailed insights into the role of galectin-3 in cardiac dysfunction and remodeling. Sharma et al. (2004) demonstrated that a 4-week continuous infusion of low-dose galectin-3 into the pericardial sac of healthy Sprague-Dawley rats resulted in left ventricular dysfunction, including impaired diastolic function [29]. This study highlighted the role of galectin-3 in marking activated macrophages in hypertrophied hearts prone to failure and contributing significantly to cardiac dysfunction. Further supporting these findings, Liu et al. (2009) investigated the effects of the peptide N-acetyl-seryl-aspartyl-lysyl-proline (Ac-SDKP) on galectin-3-induced cardiac remodeling [30]. The study revealed that Ac-SDKP prevents cardiac remodeling and dysfunction induced by galectin-3, demonstrating its potential therapeutic benefits in inhibiting the adverse effects of galectin-3 on the heart. Nguyen et al. (2018) provided additional insights into the mechanisms responsible for increased circulating levels of galectin-3 in cardiomyopathy and heart failure [31]. Their findings emphasized that galectin-3 upregulation is associated with cardiomyocyte stress and adverse cardiac remodeling, contributing to the progression of heart failure. Yu et al. (2013) explored both genetic and pharmacological inhibition of galectin-3 in preventing cardiac remodeling [32]. Their study showed that inhibition of galectin-3 interfered with myocardial fibrogenesis, thereby mitigating cardiac remodeling and improving cardiac function. This underscores the potential of targeting galectin-3 as a therapeutic strategy to prevent heart failure. In addition, investigations employing pressure-overloaded heart models revealed that stretch-induced sarcoplasmic reticulum calcium leak serves as a causal factor for atrial fibrillation, with galectin-3 inhibition exhibiting potential in mitigating doxorubicin-induced cardiac dysfunction [33,34]. Moreover, studies employing isolated subendocardial damage models unveiled alterations in myocardial microstructure and function, implicating galectin-3 in the pathophysiology of cardiac remodeling and dysfunction [35]. Notably, galectin-3 has been linked to cardiac fibrosis and inflammation, thereby contributing to the progression of diastolic dysfunction. The CT-1 (cardiotrophin-1)-Gal-3 (galectin-3) axis emerged as a pivotal player in cardiac fibrosis, shedding light on galectin-3’s role in promoting adverse myocardial remodeling and functional impairment [36]. Furthermore, evidence from cellular and animal models, coupled with clinical indices, suggests a correlation between galectin-3 levels and the severity of cardiac diastolic dysfunction [37]. Remarkably, in mice with Pkd1 (polycystic kidney disease 1) deficiency—a model associated with cardiac dysfunction—galectin-3 knockout demonstrated a rescue effect on the phenotype, underscoring its involvement in the pathogenesis of cardiac abnormalities [38]. Collectively, these findings underscore the multifaceted role of galectin-3 in the pathophysiology of diastolic dysfunction, encapsulating calcium handling abnormalities, myocardial microstructural alterations, fibrosis, and inflammation. Animal models serve as indispensable tools for elucidating these mechanisms, offering valuable insights into potential therapeutic strategies targeting galectin-3 to mitigate diastolic dysfunction and its associated complications.

### 3.2. Clinical Studies in Human Subjects

Clinical investigations provide robust evidence elucidating the involvement of galectin-3 in diastolic dysfunction across diverse patient cohorts (Table 1). In the CARE-HF trial, elevated galectin-3 levels were significantly associated with long-term cardiovascular outcomes in patients with heart failure, left ventricular dysfunction, and dyssynchrony, highlighting its role in fibrosis and adverse remodeling [39]. In elderly patients, biomarker profiling indicated that elevated galectin-3 levels were linked to the risk of developing HFpEF, suggesting a predictive value for future heart failure events [40]. In the Aldo-DHF trial, galectin-3 was found to correlate with markers of cardiac injury and stress, implying a mechanistic role in the development of diastolic dysfunction in patients with preserved ejection fraction [41]. Additionally, the DEAL-HF study demonstrated the prognostic value of galectin-3 in chronic heart failure patients, where higher levels were associated with worse outcomes, further underscoring its significance in cardiac fibrosis [42]. The DIAST-CHF study provided insights into the diagnostic and prognostic value of galectin-3 in patients at risk for HFpEF, where elevated levels were indicative of diastolic dysfunction and adverse cardiac events [43]. Moreover, data from the HF-ACTION study revealed that galectin-3 levels in ambulatory heart failure patients were predictive of long-term outcomes, reinforcing its utility as a biomarker for heart failure management [44]. A recent meta-analysis confirmed the association of galectin-3 with long-term all-cause mortality and hospitalization in heart failure patients, cementing its role as a significant prognostic marker [45]. In individuals at risk of heart failure, elevated markers of type I collagen synthesis, including galectin-3, were correlated with impaired cardiac mechanics, suggesting a potential link between fibrosis and diastolic dysfunction [46]. Similarly, in elderly patients, biomarkers of cardiac injury, stress, and fibrosis, including galectin-3, exhibited correlations with altered cardiac mechanics, implying a mechanistic role in the development of diastolic dysfunction [47]. Anemia, a prevalent comorbidity in HFpEF, has been associated with diastolic dysfunction. Clinical studies have demonstrated associations between galectin-3 levels and the severity of anemia in HFpEF patients, suggesting its potential as a biomarker for identifying diastolic dysfunction in this population [48]. Moreover, in individuals with ST-segment elevation myocardial infarction, elevated galectin-3 levels were predictive of post-infarction heart failure development, emphasizing its role in adverse cardiac remodeling and the progression of diastolic dysfunction [49]. Longitudinal investigations have provided insights into the evolution of diastolic dysfunction following myocardial infarction. Patients with anterior Q-wave myocardial infarction demonstrated progressive diastolic dysfunction over time, with galectin-3 levels predicting left ventricular remodeling and adverse outcomes post-infarction [50,51]. Furthermore, in hemodialysis patients, galectin-3 emerged as a biomarker of diastolic dysfunction, reflecting its clinical utility in assessing cardiac function in high-risk populations [52]. Overall, these clinical studies underscore the significance of galectin-3 in the pathogenesis and prognostication of diastolic dysfunction, highlighting its potential as a therapeutic target and diagnostic tool in cardiovascular disease management.

### 3.3. Galectin-3 as a Biomarker for Diastolic Dysfunction

Galectin-3 has emerged as a promising biomarker for evaluating diastolic dysfunction across diverse clinical contexts. Investigations in various patient populations consistently indicate associations between elevated galectin-3 levels and indicators of diastolic dysfunction. In individuals with chronic kidney disease (CKD), galectin-3 levels were correlated with echocardiography parameters indicative of diastolic dysfunction, suggesting its utility as a biomarker for cardiac fibrosis and dysfunction in this population [54]. Similarly, in children with end-stage renal disease undergoing regular hemodialysis, galectin-3 was identified as an early marker of diastolic dysfunction, underscoring its potential role in the early detection and monitoring of cardiac involvement in pediatric CKD [13]. Furthermore, in patients with aortic stenosis and concomitant diabetes, galectin-3 levels were associated with systemic proinflammatory and profibrotic responses, myocardial remodeling, and adverse clinical outcomes, emphasizing its involvement in the pathogenesis of diastolic dysfunction in this high-risk population [10]. Additionally, in patients with non-ischemic dilated cardiomyopathy, galectin-3 levels were correlated with circulating collagen turnover biomarkers and late gadolinium enhancement on cardiac magnetic resonance imaging, indicating its potential as a biomarker for cardiac fibrosis and adverse remodeling in this cohort [55]. Moreover, meta-analyses have highlighted the clinical implications of plasma galectin-3 levels in HFpEF, showing consistent associations with diastolic dysfunction severity and adverse outcomes [9]. Additionally, in patients with chronic Chagas cardiomyopathy, galectin-3 levels were correlated with cardiovascular biomarkers and diastolic dysfunction parameters, suggesting its potential as a prognostic marker in this specific cardiomyopathy [56].

Meanwhile, BNP (B-type natriuretic peptide), another well-established biomarker in heart failure, complements galectin-3 in assessing and managing diastolic dysfunction. Galectin-3, reflecting fibrotic processes, correlates with cardiac dysfunction severity across various conditions [57]. Concurrently, BNP, a marker of cardiac strain, complements galectin-3 by providing insights into disease progression and response to treatment [58]. Studies suggest that combining galectin-3 and BNP assessments enhances diagnostic accuracy and prognostic value, guiding personalized therapeutic strategies [59]. This dual biomarker approach not only aids in early detection of diastolic dysfunction but also supports tailored interventions to mitigate adverse outcomes [60]. Integrating galectin-3 and BNP measurements in clinical practice thus holds promise for optimizing patient management in diastolic dysfunction. Overall, these findings underscore the value of galectin-3 as a biomarker for assessing diastolic dysfunction across different patient populations, providing insights into its pathophysiological role and clinical implications in cardiovascular disease management.

## 4. Pathophysiological Insights

### 4.1. Inflammation and Fibrosis

Galectin-3 emerges as a pivotal mediator in the pathogenesis of cardiac inflammation and fibrosis, significantly impacting the development and progression of diastolic dysfunction. A comprehensive understanding of its multifaceted role in cardiac pathology has been elucidated through recent investigations. Seropian et al. (2023) delineated the involvement of galectin-3 in cardiac dysfunction and toxicity [61], particularly in doxorubicin-treated murine models, where augmentation of galectin-3 correlated with heightened oxidative stress and fibrotic progression, thus exacerbating myocardial injury. Moreover, Wang et al. (2023) showcased the therapeutic potential of targeting galectin-3 post-infarction [62], demonstrating its efficacy in attenuating progressive fibrosis by modulating inflammatory profibrotic pathways, thereby preserving diastolic function following myocardial infarction. Furthermore, Hu et al. (2023) unraveled a galectin-3-centered paracrine network orchestrating cardiac inflammation and fibrosis in response to beta-adrenergic insult [63], underscoring its pivotal role in cardiac remodeling. Genetic studies have further underscored the significance of galectin-3, as reported by Fontana Estevez et al. (2022), wherein genetic deletion exacerbated age-related myocardial hypertrophy and fibrosis [64], suggesting a protective role against pathological remodeling. Conversely, Vlachou et al. (2022) demonstrated the detrimental impact of galectin-3 interference in promoting cardiac dysfunction and comorbidities in a genetic heart failure model [65], indicating a disruption in tissue repair mechanisms and functional recovery. These collective findings underscore the indispensable role of galectin-3 in mediating cardiac inflammation and fibrosis, thereby contributing to the pathogenesis of diastolic dysfunction and adverse cardiac remodeling. Targeting galectin-3 signaling pathways holds significant promise as a therapeutic strategy for managing diastolic dysfunction and enhancing clinical outcomes in heart failure.

### 4.2. Cardiac Remodeling

Cardiac remodeling, a multifaceted process encompassing structural and functional alterations in response to pathological stimuli, encompasses diastolic dysfunction, a hallmark feature of HFpEF. Galectin-3 emerges as a pivotal player in cardiac remodeling, exerting profound effects on the progression of diastolic dysfunction and HFpEF. Experimental evidence highlights the detrimental impact of galectin-3 on cardiac structure and function. Seropian et al. (2023) delineated its role in acute cardiac dysfunction and toxicity, primarily through mechanisms involving heightened oxidative stress and fibrosis [61]. This pro-fibrotic action of galectin-3 exacerbates myocardial stiffness and impairs diastolic relaxation, thereby promoting diastolic dysfunction. Moreover, inhibition of galectin-3 holds promise in attenuating cardiac fibrosis and remodeling. Wang et al. (2023) demonstrated the efficacy of post-infarction galectin-3 inhibition in mitigating progressive fibrosis by modulating inflammatory profibrotic cascades [62], offering potential therapeutic avenues for preserving diastolic function in various cardiac pathologies. Genetic studies further elucidate the significance of galectin-3 in cardiac remodeling. Fontana Estevez et al. (2022) demonstrated that genetic deletion exacerbates age-related myocardial hypertrophy and fibrosis [66], highlighting its protective role against pathological cardiac remodeling. In a heart failure model, Sonkawade et al. (2021) explored the therapeutic potential of small endogenous peptides in mitigating myocardial remodeling induced by galectin-3 overexpression [67], underscoring the promise of galectin-3 targeting for modulating pathological cardiac remodeling and improving diastolic function. Collectively, these studies underscore the critical role of galectin-3 in mediating cardiac fibrosis and diastolic dysfunction, highlighting its potential as a therapeutic target for managing HFpEF and other cardiac conditions characterized by impaired diastolic function.

### 4.3. Endothelial Dysfunction

Endothelial dysfunction, characterized by impaired vascular homeostasis and endothelial barrier integrity, contributes significantly to the pathogenesis of diastolic dysfunction and cardiovascular diseases. Galectin-3 emerges as a central mediator in endothelial dysfunction, exacerbating vascular inflammation and oxidative stress. Recent investigations have provided insights into the role of galectin-3 in mediating endothelial dysfunction across various cardiovascular conditions. Wang et al. (2024) elucidated its involvement in inflammation and fibrosis in arteriogenic erectile dysfunction, highlighting its pro-inflammatory role via the toll-like receptor 4 (TLR4)/myeloid differentiation primary response 88 (MyD88)/NF-kappaB pathway [68]. Additionally, Pang et al. (2023) identified Yes-associated protein (YAP)-galectin-3 signaling as a mediator of endothelial dysfunction in angiotensin II-induced hypertension, emphasizing the detrimental effects of galectin-3 on vascular homeostasis and endothelial function [69]. Exploring the prognostic implications, Tsigkou et al. (2023) investigated the role of galectin-3 and endothelial function in heart failure [70], suggesting a potential link between galectin-3-mediated endothelial dysfunction and adverse cardiovascular outcomes. Mechanistically, galectin-3 exacerbates endothelial injury by inducing inflammation and oxidative stress, as demonstrated by Chen et al. (2019) [71], highlighting its role in promoting vascular inflammation and dysfunction. Overall, galectin-3-mediated endothelial dysfunction contributes significantly to the pathogenesis of diastolic dysfunction and cardiovascular diseases, underscoring its potential as a therapeutic target for mitigating vascular inflammation and improving endothelial function.

## 5. Diagnostic and Therapeutic Implications

### 5.1. Potential Diagnostic Utility of Galectin-3

Galectin-3 emerges as a promising diagnostic biomarker for diastolic dysfunction, reflecting its involvement in diverse underlying pathophysiological processes. Consistent findings link elevated galectin-3 levels to heightened cardiovascular event risk and heart failure incidence, underscoring its diagnostic potential [72,73,74,75]. Furthermore, galectin-3 levels correlate with disease severity and prognosis in heart failure and atrial fibrillation, offering valuable insights for risk stratification and treatment decision making [73,75,76]. Integrating galectin-3 measurements into clinical practice may refine diagnostic accuracy and facilitate tailored management strategies for cardiovascular diseases. Galectin-3 assessment holds promise for early detection and risk stratification in diastolic dysfunction patients, furnishing crucial prognostic information. Incorporating galectin-3 testing into diagnostic protocols for HFpEF could refine risk assessment and guide therapeutic interventions.

### 5.2. Therapeutic Targeting of Galectin-3

Therapeutic intervention targeting galectin-3 emerges as a promising avenue for managing diastolic dysfunction and its associated cardiovascular complications (Table 2). However, Gandhi et al. (2015) found that mineralocorticoid receptor antagonist therapy did not provide significant benefit for patients with heart failure and elevated galectin-3 levels [77]. Despite mineralocorticoid receptor antagonists’ role in reducing fibrosis through aldosterone blockade, high galectin-3 levels, indicative of a complex fibrotic process, might involve pathways that mineralocorticoid receptor antagonists do not target. This suggests the need for alternative therapies to address the multifaceted mechanisms of fibrosis in these patients. Ongoing efforts focus on the development of drugs aimed at modulating galectin-3 activity to counter its pathological effects [78]. Encouragingly, preclinical studies demonstrate the efficacy of galectin-3 inhibition in mitigating inflammation and fibrosis, hinting at its therapeutic potential [79]. Furthermore, the prospect of targeting galectin-3 presents a novel therapeutic strategy for cardiovascular diseases by mitigating cardiac fibrosis, hypertrophy, and inflammation [72,80]. These insights underscore the therapeutic promise of galectin-3 inhibition in addressing the underlying mechanisms of cardiovascular pathologies, thus enhancing clinical outcomes. Clinical trials are actively investigating the safety and efficacy of galectin-3 inhibitors in heart failure patients, particularly those with preserved ejection fraction. The targeted modulation of galectin-3 signaling pathways represents a promising therapeutic approach for diastolic dysfunction, offering a complementary strategy to existing treatments.

### 5.3. Future Directions and Research Opportunities

Future research avenues in galectin-3 and diastolic dysfunction encompass elucidating molecular mechanisms, validating diagnostic and prognostic utility, developing novel therapeutics, and exploring galectin-3 as a biomarker for monitoring disease progression and treatment response. Prospective clinical studies are needed to standardize diagnostic algorithms and risk stratification models. Collaborative efforts across disciplines will expedite the translation of research findings into clinical practice, ultimately improving patient outcomes and advancing cardiovascular care.

## 6. Conclusions

### 6.1. Summary of Key Findings

**Figure 3:**
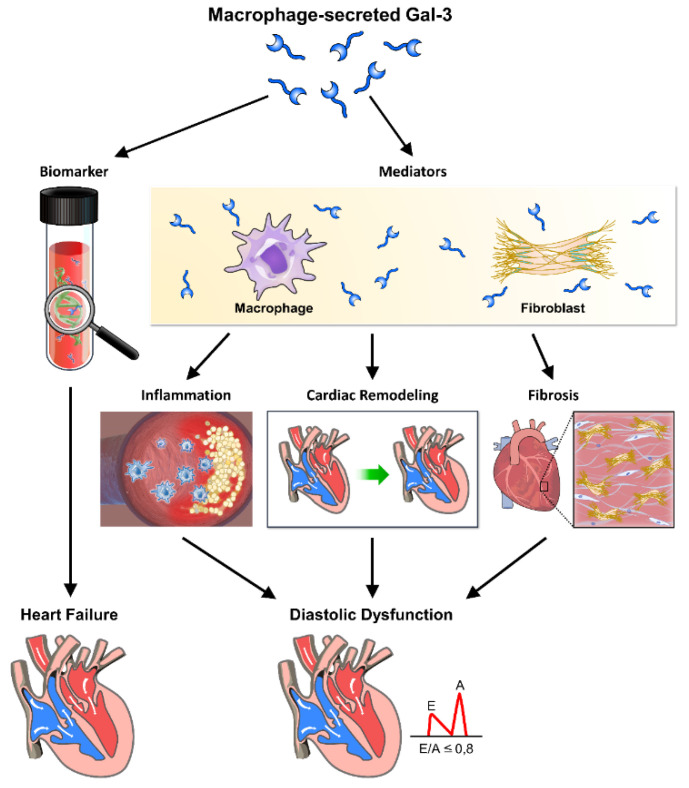
The role of galectin-3 in inflammation, fibrosis, and heart remodeling leading to cardiac diastolic dysfunction.

In this comprehensive review, we have meticulously examined the intricate interplay between galectin-3 and diastolic dysfunction, illuminating its multifaceted role in the pathophysiology of HFpEF. Our analysis reveals that galectin-3 functions both as a biomarker and mediator, orchestrating inflammation, fibrosis, and cardiac remodeling processes that drive the development and progression of diastolic dysfunction (Figure 3). Through its modulation of cellular signaling pathways and interactions with extracellular matrix components, galectin-3 profoundly impacts myocardial stiffness, vascular function, and endothelial integrity. Clinical investigations underscore its diagnostic and prognostic value in identifying high-risk patients and guiding therapeutic strategies. Moving forward, further research is imperative to dissect the precise mechanisms underpinning galectin-3-mediated pathophysiology and to explore its therapeutic potential in ameliorating diastolic dysfunction and enhancing patient outcomes in HFpEF.

Galectin-3 (Gal-3), secreted by macrophages, plays a crucial role in the progression of cardiac diastolic dysfunction through its involvement in inflammation, fibrosis, and heart remodeling. This figure illustrates the pathways and mechanisms by which Gal-3 contributes to these pathological processes, ultimately leading to heart failure. Through these mechanisms, galectin-3 significantly contributes to the pathophysiology of cardiac diastolic dysfunction, highlighting its potential as a therapeutic target and diagnostic marker. Macrophage-secreted Gal-3: galectin-3 released by macrophages acts as a key mediator in the inflammatory response. Biomarker: galectin-3 serves as a biomarker for inflammation and fibrosis in cardiac tissues. Mediators: various inflammatory mediators are regulated by Gal-3, contributing to cardiac inflammation. Inflammation: Gal-3 promotes inflammation, which exacerbates cardiac tissue damage and remodeling. Cardiac remodeling: structural changes in the heart induced by Gal-3, including hypertrophy and fibrosis, impair cardiac function. Fibrosis: Gal-3 stimulates fibroblast activation and collagen deposition, leading to increased stiffness and fibrosis of the cardiac tissue. Heart failure: the combined effects of inflammation, fibrosis, and remodeling contribute to the development of heart failure. Diastolic dysfunction: impaired relaxation and filling of the heart during diastole, driven by Gal-3-mediated pathological changes.

### 6.2. Clinical Relevance and Implications

Galectin-3 holds significant clinical relevance in diastolic dysfunction (Table 3), serving as a promising diagnostic biomarker and therapeutic target for HFpEF. By elucidating its pivotal role in inflammation, fibrosis, and cardiac remodeling, our review underscores galectin-3’s utility as an indicator of disease severity and prognosis in diastolic dysfunction patients (Table 4). Moreover, therapeutic interventions targeting galectin-3 offer a novel avenue for attenuating myocardial stiffness, improving vascular function, and preserving endothelial integrity, thereby mitigating disease progression and reducing HFpEF-related morbidity and mortality. Recognizing galectin-3 as a clinically relevant marker and therapeutic avenue underscores the importance of personalized management approaches tailored to the underlying pathophysiology of diastolic dysfunction, ultimately enhancing patient care and outcomes in HFpEF.

### 6.3. Closing Remarks on the Role of Galectin-3 in Diastolic Dysfunction

In conclusion, our comprehensive investigation into galectin-3’s role in diastolic dysfunction unveils its multifaceted involvement in the pathophysiological mechanisms driving HFpEF. Galectin-3 emerges not only as a diagnostic biomarker but also as a therapeutic target with promising implications for clinical outcomes in diastolic dysfunction patients. In HFpEF, galectin-3 levels are often elevated, correlating with the extent of left ventricular systolic dysfunction and adverse remodeling. This biomarker is associated with increased mortality and hospitalizations, as it reflects ongoing myocardial fibrosis and inflammation [77]. Mineralocorticoid receptor antagonists, which reduce fibrosis, have been found to be particularly beneficial in patients with high galectin-3 levels, highlighting the protein’s role in therapeutic stratification [77]. In contrast, in HFpEF, galectin-3’s utility appears more nuanced. HFpEF is characterized by diastolic dysfunction, where myocardial stiffness and impaired relaxation predominate. Galectin-3 contributes to the pathophysiology by promoting fibrosis and inflammation within the myocardium, exacerbating diastolic dysfunction [5,37]. Elevated galectin-3 levels in HFpEF patients correlate with worse outcomes and can serve as an indicator of disease severity and progression [9]. However, therapeutic interventions targeting galectin-3 in HFpEF are less well-defined, necessitating further research to explore potential benefits. Overall, while galectin-3 is a promising biomarker in both HFrEF and HFpEF, its role and therapeutic implications differ, warranting tailored approaches to management in these distinct heart failure phenotypes. By delineating its contributions to inflammation, fibrosis, cardiac remodeling, and endothelial dysfunction, our review underscores galectin-3’s significance in disease progression and highlights its potential as a target for innovative therapeutic interventions aimed at alleviating myocardial stiffness and preserving vascular function. Embracing galectin-3 as a pivotal player in the pathogenesis of diastolic dysfunction opens avenues for personalized treatment strategies tailored to address the underlying molecular mechanisms, ultimately paving the way for improved management and prognosis in HFpEF patients.

## Figures and Tables

**Table 1:** Clinical galectin-3 studies in diverse patient cohorts with diastolic dysfunction.

Title	Authors	Years	Results
Galectin-3 as an early marker of diastolic dysfunction in children with end-stage renal disease on regular hemodialysis.	Akram et al. [13]	2022	Galectin-3 is a potential early biomarker that can be used in early diagnosis and grading of diastolic dysfunction in end-stage renal disease children on regular hemodialysis.
Impact of diabetes on serum biomarkers in heart failure with preserved ejection fraction: insights from the TOPCAT trial.	De Marco et al. [28]	2021	Higher galectin-3 levels were measured in patients with HFpEF.
The diagnostic and prognostic value of galectin-3 in patients at risk for heart failure with preserved ejection fraction: results from the DIAST-CHF study.	Trippel et al. [43]	2021	Galectin-3 differentiated patients with HFpEF from an overall cohort of well-characterized patients with risk factors for HFpEF.
Cardiac remodeling biomarkers as potential circulating markersofleft ventricular hypertrophy in heart failure with preserved ejection fraction.	Mitic et al. [53]	2020	Cardiac remodeling biomarkers (e.g., galectin-3) are potential circulating indicators of left ventricular hypertrophy in HFpEF, which may ensure timely recognition of disease progression among high-risk patients.
Clinical, demographic, and imaging correlates of anemia in heart failure with preserved ejection fraction (from the RELAX Trial).	Parcha et al. [48]	2020	Galectin-3 levels were higher in anemic HFpEF patients.
Echocardiographic diastolic function evolution in patients with an anterior Q-wave myocardial infarction: insights from the REVE-2 study.	Ferreira et al. [50]	2019	The amino-terminal propeptide of type III procollagen, galectin-3, and BNP may be independently associated with new-onset diastolic dysfunction in post- myocardial infarction patients.
Galectin-3 predicts left ventricular remodeling after anterior-wall myocardial infarction treated by primary percutaneous coronary intervention.	Di Tano et al. [51]	2017	Left ventricular end-diastolic volume and galectin-3 levels independently predicted left ventricular remodeling.
Galectin-3 as a new biomarker of diastolic dysfunction in hemodialysis patients.	Gurel et al. [52]	2015	Galectin-3 may be a promising biomarker for the detection of left ventricular diastolic dysfunction in hemodialysis.

This table synthesizes the findings from various clinical studies, demonstrating the role of galectin-3 in different aspects of diastolic dysfunction and heart failure across diverse patient populations.

**Table 2:** Summary of preclinical and clinical development of therapeutic inhibitors of galectin-3.

Therapeutic Inhibitor	Development Stage	Methodology	Key Findings	Conclusion	Reference
GM-CT-01 (galactoarabino-rhamnogalacturonate)	Preclinical	Animal models	GM-CT-01 inhibited tumor growth and metastasis in mice by targeting galectin-3	GM-CT-01 has potential for cancer therapy targeting galectin-3	Henderson et al. (2006) [81]
Modified citrus pectin (MCP)	Preclinical	Rat models of hypertension-induced heart failure	MCP treatment resulted in reduced cardiac hypertrophy and fibrosis	MCP is a promising agent for treating hypertension-induced cardiac complications	Calvier et al. (2013) [82]
Modified citrus pectin (MCP)	Preclinical	Animal models and in vitro studies	MCP reduced myocardial fibrogenesis and improved cardiac function by inhibiting galectin-3	MCP shows potential as a therapeutic agent for cardiac remodeling	Yu et al. (2013) [32]
Belapectin (GR-MD-02)	Phase II clinical trial	Patients with NASH and advanced fibrosis	Belapectin significantly reduced liver fibrosis in patients	Belapectin is a promising therapeutic for advanced fibrosis in NASH patients	Chalasani et al. (2020) [83]
TD139 (inhaled galectin-3 inhibitor)	Phase I clinical trial	Healthy volunteers	TD139 was well-tolerated with no significant adverse effects, demonstrating potential for inhaled delivery	TD139 shows promise for treating lung diseases mediated by galectin-3	Hirani et al. (2021) [84]
PectaSol-C (modified citrus pectin)	Phase II clinical trial	Human clinical trials, animal models	MCP significantly reduced metastasis and tumor growth in prostate and breast cancer models. In clinical settings, MCP improved patient outcomes with minimal side effects.	MCP shows promise as an effective therapeutic inhibitor of Galectin-3 in cancer treatment. Further large-scale clinical trials are needed to confirm its efficacy.	Keizman et al. (2023) [85]

This table summarizes the current state of preclinical and clinical development of therapeutic inhibitors targeting galectin-3, a protein implicated in fibrosis and inflammation. This comprehensive overview highlights the potential of galectin-3 inhibitors in treating fibrotic diseases and improving clinical outcomes in conditions such as chronic heart failure, idiopathic pulmonary fibrosis, and nonalcoholic steatohepatitis (NASH).

**Table 3:** Summary of key studies on galectin-3 and diastolic dysfunction.

Study	Population	Key Findings	Conclusion
Bellos et al. (2024) [86]	Hemodialysis patients	Elevated serum galectin-3 levels are associated with higher mortality and cardiovascular outcomes.	Galectin-3 is a significant prognostic marker in hemodialysis patients.
Spahillari et al. (2024) [87]	Heart failure patients	MicroRNAs associated with cardiac biomarkers, structure, function, and incident outcomes.	Galectin-3 correlates with cardiac remodeling and outcomes in heart failure.
Winter et al. (2023) [88]	Dogs with pulmonary stenosis	Higher circulating galectin-3 levels linked to right ventricular diastolic and systolic dysfunction.	Galectin-3 can be a biomarker for cardiac function in canine models.
Ureche et al. (2023) [54]	Advanced CKD patients	Cardiac fibrosis biomarkers, including galectin-3, correlate with echocardiographic parameters.	Galectin-3 is linked to cardiac fibrosis and diastolic dysfunction in CKD patients.
Baccouche et al. (2023) [5]	HFpEF patients	Galectin-3 associated with HFpEF.	Galectin-3 is an emerging marker in HFpEF.
Lee et al. (2023) [10]	Aortic stenosis patients with diabetes	Proinflammatory-profibrotic response associated with myocardial remodeling and clinical outcomes.	Galectin-3 contributes to cardiac remodeling in diabetic aortic stenosis patients.
Elsadek et al. (2022) [13]	Children with end-stage renal disease	Early increase in galectin-3 levels noted in children with diastolic dysfunction on hemodialysis.	Galectin-3 as an early marker for diastolic dysfunction in pediatric renal disease.
Kondratavičienė et al. (2022) [89]	Obstructive sleep apnea patients	Treatment with continuous positive airway pressure (CPAP) improved left heart geometry, function, and reduced galectin-3 levels.	Galectin-3 reduction linked to improved cardiac function post-CPAP treatment.
Revnic et al. (2022) [55]	Non-ischemic dilated cardiomyopathy (DCM) patients	Galectin-3 levels correlated with cardiac function and fibrosis markers.	Galectin-3 is a predictive biomarker for cardiac dysfunction in non-ischemic DCM.
Kobayashi et al. (2022) [46]	Heart failure patients	Markers of type I collagen synthesis, including galectin-3, predict response to spironolactone.	Galectin-3 as a predictor for therapeutic response in heart failure.
Shi et al. (2022) [9]	HFpEF patients	Meta-analysis showing significant association between galectin-3 and HFpEF outcomes.	Galectin-3 is a valuable biomarker for HFpEF prognosis.
Karolko et al. (2022) [90]	Patients with exertional dyspnea	Moderately reduced renal function impacts the diagnostic and prognostic value of galectin-3.	Renal function must be considered when evaluating galectin-3 levels.
Vlachou et al. (2022) [65]	Genetic heart failure model	Galectin-3 promotes cardiac dysfunction and comorbidities by interfering with tissue repair.	Targeting galectin-3 may help mitigate heart failure progression.

This table consolidates findings from diverse populations, highlighting the broad relevance of galectin-3 in predicting and understanding diastolic dysfunction across different clinical settings.

**Table 4:** Comparison of galectin-3 with other biomarkers for diastolic dysfunction.

Biomarker	Mechanism of Action/Role	Clinical Significance in Diastolic Dysfunction	References
Galectin-3	Modulates fibrosis and inflammation by binding to β-galactosides on cell surfaces and extracellular matrix proteins.	Elevated levels are associated with heart failure, myocardial fibrosis, and poor outcomes in patients with diastolic dysfunction.	Baccouche and Rhodenhiser, 2023 [5]
NT-proBNP	Released in response to ventricular stretching and pressure overload.	High levels indicate heart failure and correlate with severity of diastolic dysfunction.	Spahillari et al., 2024 [87]
sST2	A member of the interleukin-1 receptor family that modulates immune response.	Elevated levels are indicative of myocardial stress and fibrosis, predicting adverse outcomes in diastolic dysfunction.	Elsadek et al., 2022 [13]
GDF-15	A member of the TGF-β cytokine family, involved in inflammation and apoptosis.	Increased levels are linked to myocardial infarction, heart failure, and diastolic dysfunction severity.	Węgiel et al., 2022 [91]
Collagen Turnover Markers	Indicators of collagen synthesis and degradation in the extracellular matrix.	Elevated in conditions leading to fibrosis, these markers correlate with severity and progression of diastolic dysfunction.	Kobayashi et al., 2022 [46]
MiRNAs	Small non-coding RNAs that regulate gene expression post-transcriptionally.	Specific miRNAs are associated with cardiac fibrosis, hypertrophy, and diastolic dysfunction.	Spahillari et al., 2024 [87]
VAP-1	Enzyme involved in inflammation and leukocyte migration.	Higher levels predict cardiovascular events and are associated with endothelial dysfunction in diastolic heart failure.	Kim et al., 2021 [92]

Galectin-3 is recognized as a significant biomarker in the context of diastolic dysfunction due to its role in modulating fibrosis and inflammation. Its elevated levels are consistently associated with worse clinical outcomes in heart failure patients, particularly those with preserved ejection fraction. Compared to other biomarkers such as N-terminal prohormone of brain natriuretic peptide (NT-proBNP), soluble suppression of tumorigenesis-2 (sST2), growth differentiation factor 15 (GDF-15), collagen turnover markers [e.g., type I procollagen carboxyterminal propeptide (PICP), C-telopeptide for type I collagen (CITP)], specific microRNAs (miRNAs), and vascular adhesion protein-1 (VAP-1), galectin-3 provides unique insights into myocardial fibrosis and remodeling processes, making it a valuable tool in the assessment and management of diastolic dysfunction. This table synthesizes data from multiple studies, providing a comparative overview of the clinical utility of various biomarkers in diastolic dysfunction, emphasizing galectin-3’s prominent role.

## Data Availability

Not applicable.
